# Gender Different Response to Immunonutrition in Liver Cirrhosis with Sepsis in Rats

**DOI:** 10.3390/nu4030231

**Published:** 2012-03-22

**Authors:** Tsann-Long Hwang, Chi-Yi Chen

**Affiliations:** Department of Surgery, Chang Gung Memorial Hospital, Chang Gung University, Tao-Yuan 333, Taiwan; Email: yiiyii@adm.cgmh.org.tw

**Keywords:** gender difference, immunonutrition, liver cirrhosis, sepsis, immunity, cecal ligation and puncture, sex hormone

## Abstract

Females with sepsis have a better prognosis than males, while those of both genders with cirrhosis have a high mortality. Impaired immunity accompanies liver cirrhosis. The potential association between sex and immunologic response of cirrhotic rats in sepsis following immunonutrition was investigated. One hundred and forty-three rats were randomly divided into groups. Liver cirrhosis was produced by weekly feeding of CCl_4_ for 8 weeks. Among them, 24 male and 19 female underwent castration one month before studying. The rats were fed with either immune enhancing diet or control diet for five days, then sepsis was induced with cecal ligation and two holes puncture. Main outcomes included mortality and serum cytokines (IL-1β, 6, and 10). Comparisons were made both within and between genders. Cirrhotic non-castrated male rats showed a significant decrease in mortality (64.1% *vs.* 32.1%, *p* = 0.032) with better survival than control diet following immune enhancing diet. Lower mortality of cirrhotic non-castrated female rats was found after immune enhancing diet (69.6% *vs.* 52.1%, *p* = 0.365). Cirrhotic castrated male rats showed a lower mortality (44.4%) following immune enhancing diet, and cirrhotic castrated female rats also showed significantly lower mortality and better survival than control diet after immune enhancing diet (87.5% *vs.* 33.3%, *p* = 0.004). Plasma concentrations of IL-1β were higher in non-oophorectomized female rats fed with control diet compared to immune enhancing diet. Non-orchidectomized males and non-oophorectomized females exhibited similar increases in IL-10 after immune enhancing diet. Our results demonstrated that immunonutrition was more beneficial for male than female cirrhotic rats following sepsis. Though orchidectomy was not found to be more advantageous for the normal male rats in sepsis, immunonutrition seemed to be as important as sex hormone for female rats in sepsis.

## 1. Introduction

Behind heart disease and cancer, cirrhosis is the third most common cause of death among people aged 45 to 65 years. Almost 2% of the western population has cirrhosis. Many people with mild cirrhosis usually have no symptoms and appear to be well for years. Such cases are usually discovered during routine tests for other problems or during surgery. But, if patients have moderate to severe cirrhosis of the liver, they do not possess enough healthy liver tissue to perform the metabolic and detoxification processes that keep the body healthy, especially at times of stress or sepsis [1,2].

Gender differences have been reported following trauma and sepsis; female patients have a better outcome than males, which may relate to increased levels of anti-inflammatory cytokines [3,4,5,6,7,8]. Several studies [9,10,11] have demonstrated gender differences in susceptibility to septic challenge. In female mice, following cecal ligation and puncture (CLP), improved cell-mediated immune response resulted in a significantly improved ability to tolerate sepsis than males [11]. Sex steroids may contribute to the above observations regarding sexual dimorphism, and androgens have been found to react immunosuppressively [12,13,14,15,16,17]. Sexual dimorphism may be influenced by the effects of sex hormones and a different ratio of pro-inflammatory and anti-inflammatory cytokines in women compared to men [7]. Enhanced immune response in females may result from the absence of androgenic hormones in females or the immunostimulating effects of female sex steroids [18]. 

Based on the previous animal experimental and clinical studies, a clear association between sexual dimorphism and various immune functions has been proposed [11,12]. Modulation of the hormonal response to immunonutrition [12] has been demonstrated to be effective in septic complication in animal studies. We reported that immunonutrition was more important for male rats than female rats during sepsis in our previous study [11]. However, data are not yet available on the cirrhotic changes of the liver in the animal and the clinical relevance of sex-specific phenomena for immunomodulational changes after feeding septic animals or patients with immune enhancing diet. Therefore, this study attempts to assess and compare the potential association between sex and immunologic response in cirrhotic rats in sepsis following immunonutrition. 

## 2. Materials and Methods

### 2.1. Animal Study

One hundred and sixty-nine Sprague-Dawley rats weighing 150–200 g were used in the study, including 89 males and 80 females. All animals received humane care and the study protocols were compliant with the guidelines of Chang Gung Memorial Hospital. The animals were fed in cages for at least for five days before the experiment, and diseased rats with body weight loss were excluded. All animals were fasted overnight with free access to water before the study. 

### 2.2. Production of Liver Cirrhosis

Liver cirrhosis was produced with weekly feeding of carbon tetrachloride (CCl_4_) in drinking water for 2 months. The rats were then used for further studying.

### 2.3. Estrous Phase of Female Rat Was Proven

Female rats underwent vaginal smear examinations to determine the stage of the estrous cycle. The day of estrous was characterized by large clumps of cornified cells. Female rats were chosen based on their estrous phase, determined by vaginal smear examination: Low levels of circulating female hormones were chosen at the beginning of diet feeding, rising to high levels at the end [19].

### 2.4. Grouping and Studying Procedures

All 102 rats were divided as follows. Fifty-six cirrhotic males were divided into (1) Immune enhancing group (*n* = 28), fed with an immune enhancing diet (Nu-immune, Nutritec-Enjoy Nutrition Center, Taipei, Taiwan) for five days, then sepsis was induced by CLP; (2) Control group (*n* = 28) was fed with control diet for five days, then sepsis was induced by CLP. The other 46 cirrhotic female rats were divided into (1) Immune enhancing group (*n* = 23), fed with immune enhancing diet (Nu-immune) for five days, then sepsis was induced by CLP; (2) Control group (*n* = 23), fed with control diet for five days, then sepsis was induced by CLP.

Another 67 cirrhotic rats, including 33 males and 34 females, were fed in cages for at least five days before the experiment. Diseased rats with body weight loss were excluded. All animals were fasted overnight with free access to water prior to orchidectomy or oophorectomy. The rats underwent general anesthesia with ether before surgery, with male rats receiving bilateral orchidectomy and female rats receiving oophorectomy following laparotomy. All rats were returned to their cages after surgery and allowed a chow diet for one month. The rats were then divided as follows:

Thirty-three orchidectomized cirrhotic male rats were divided into (1) An immune enhancing group (*n* = 18), fed with immune enhancing diet (Nu-immune) for five days, and sepsis was induced by CLP; (2) A control group (*n* = 15), fed with control diet for five days, and sepsis was induced by CLP. Thirty-four oophorectomized cirrhotic female rats were divided into (1) An immune enhancing group (*n* = 18), fed with immune enhancing diet (Nu-immune) for five days, and sepsis was induced by CLP; (2) A control group (*n* = 16), fed with control diet for five days, and sepsis was induced by CLP.

The rats received five days with either an: immune enhancing diet (Nu-Immune) feeding, which contained 11.1 g of glutamine and 11.6 g of arginine in each 1000 Kcal diet; or a non-immune enhancing control diet. The contents of both diets are listed in Table 1. Sepsis then was then induced in the rats using CLP. 

The cytokines (IL-1β, 6, and 10) responses were measured using the serum of non-orchidectomized male and non-oophorectomized female rats at 0, 2, 4, 8, 12, 16 and 24 h after surgery. Comparisons were made both within and between genders. 

**Table 1:** Summary of the compositions of immune enhancing diet and control diet.

	Immune enhancing diet (IED)	Control diet
**Protein (% and source)**	22% (Whey)	14% (Whey)
l-Arginine (g/1000 Kcal)	11.6	0
l-Glutamine (g/1000 Kcal)	11.1	0
**Carbohydrate (% and source)**	53% (Maltodextrin, Sucrose)	52% (Maltodextrin)
**Fat (% and source)**	25% (Canola oil and MCT)	34% (Canola oil)
% Fatty acid polyunsaturated	65.3%	69.1%
ω-6:ω-3 Fatty acid ratio	3.3:1	3.8:1
**Vitamins & Trace elements**		
Vitamin A (mg/100 g)	591	394
Vitamin B_6_ (mg/100 g)	1.27	0.75
Vitamin B_12_ (μg/100 g)	3.6	2.3
Vitamin C (mg/100 g)	127	30
Vitamin E (mg/100 g)	25	15
Sodium (mg/100 g)	316	263
Potassium (mg/100 g)	443	575
Calcium (mg/100 g)	390	275
Magnesium (mg/100 g)	127	100
Iron (mg/100 g)	5.9	4.5
Copper (mg/100 g)	0.6	0.5
Manganese (mg/100 g)	1.7	1.3
Zinc (mg/100 g)	7.6	5.6
Selenium (mg/100 g)	27.0	21.3
**Osmolality (mOsmol/kg)**	430	300

### 2.5. Induction of Sepsis with CLP Procedure

Sepsis was induced by CLP procedure as described by Wichterman [20]. The cecum was ligated with 3-0 silk ligature and two punctures were made with an 18-gauge needle. The cecum was returned to the peritoneal cavity and the abdomen closed in two layers. All rats were resuscitated with normal saline (4 mL/100 g of body weight) both immediately following and 7 h after surgery. The rats were fasted but had free access to water. 

### 2.6. Assays of Cytokines

Commercially available ELISA-kits were used to determine interleukin IL-1β, and IL-6 in supernatants of whole blood cultures or serum specimens based on the instructions of manufacturer. The assay sensitivity, 5 pg/mL IL-10, was measured using a specific enzyme-linked immunosorbent assay, which uses the rat anti-human IL-10 monoclonal antibody and a rabbit anti-human IL-10 polyclonal antibody. 

### 2.7. Statistical Analysis

The results were presented as mean ± standard error of the mean (SEM). Chi-square analysis was used to compare the mortality of rats. One-way analysis of variance and one-way analysis of variance on rank were used to determine the significance of the differences between experimental means of changes in cytokines. *P *value less than 0.05 is considered statistically significant. 

## 3. Results

The mortalities of non-castrated rats were compared both within and between genders. Table 2 compares the mortality of cirrhotic male and female rats. CLP mortality decreased from 64.3% to 32.1% (*p* < 0.05) in cirrhotic male rats following feeding with an immune enhancing diet. In comparison, the cirrhotic female rats maintained rather similar mortality following feeding with an immune enhancing diet (69.6% *vs.* 52.1%). The mortality induced by CLP in cirrhotic male rats following feeding with non-immune enhancing control diet showed no difference to that in the cirrhotic female rats. These results demonstrated the important role of immunonutrition for cirrhotic male rats during sepsis.

**Table 2:** Comparison of mortality between different diets in cirrhotic male and cirrhotic female rats.

Mortality	Immune enhancing diet (IED)	Control diet	*p* value
Cirrhotic male rats after cecal ligation and puncture (CLP)	9/28 (32.1%)	18/28 (64.3%)	0.032
Cirrhotic female rats after CLP	12/23 (52.1%)	16/23 (69.6%)	0.365

The mortalities of castrated rats were compared both within and between genders. Table 3 compares the mortality of cirrhotic castrated male and female rats. The mortality in orchidectomized cirrhotic male rats significantly decreased from 86.7% to 44.4% following feeding with an immune enhancing diet. The mortality for oophorectomized female rats was also lower after feeding with an immune enhancing diet (87.5% *vs.* 33.3%). These results demonstrated the important role of immunonutrition for both cirrhotic castrated male and female rats during sepsis.

**Table 3:** Comparison of mortality between different diets in cirrhotic male and female rats after castration.

Mortality	Immune enhancing diet (IED)	Control diet	*p* value
Castrated cirrhotic male rats after cecal ligation and puncture (CLP)	8/18 (44.4%)	13/15 (86.7%)	0.032
Castrated cirrhotic female rats after CLP	6/18 (33.3%)	14/16 (87.5%)	0.004

**Figure 1:**
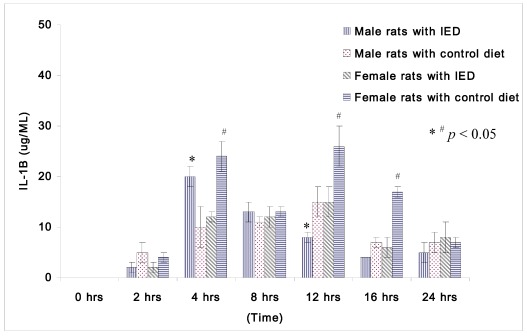
Plasma concentrations of IL-1β (μg/mL) in rats at 0, 2, 4, 8, 12, 16, and 24 h after CLP in cirrhotic female rats fed with control diet (*n* = 28), cirrhotic female rats fed with immune enhancing diet (*n* = 28), cirrhotic male rats fed with control diet (*n* = 23), and cirrhotic male rats fed with immune enhancing diet (*n* = 23). * *p* < 0.05 between male rats; ^# ^*p* < 0.05 between female rats; IL-1β, interleukin-1β. The data are expressed as Mean ± SEM.

Plasma concentrations of IL-1β at 0 h after CLP were too low to be compared among non-orchidectomized male rats and non-oophorectomized female rats. Figure 1 displays the changes in IL-1β following induction of sepsis among non-orchidectomized male rats and non-oophorectomized female rats. The female rats fed with control diet displayed significantly higher IL-1β than the female rats fed with immune enhancing diet at 4 h (24 ± 3 μg/mL *vs.* 12 ± 1 μg/mL), at 12 h (26 ± 4 μg/mL *vs.* 15 ± 3 μg/mL), and at 16 h (17 ± 1 μg/mL *vs.* 6 ± 2 μg/mL) after CLP. However, the male rats with immune enhancing diet had higher IL-1β than the rats with control diet at 4 h (20 ± 2 μg/mL *vs.* 10 ± 4 μg/mL), but lower levels of IL-1β at 12 h (8 ± 1 μg/mL *vs.* 15 ± 3 μg/mL) after CLP. 

**Figure 2:**
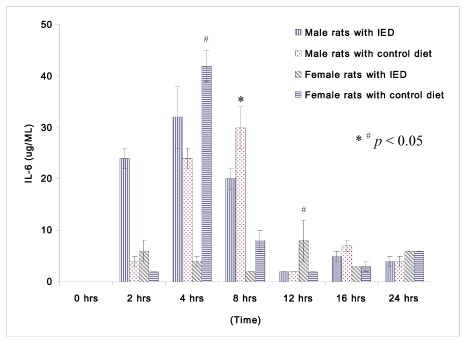
Plasma concentrations of IL-β (μg/mL) in rats at 0, 2, 4, 8, 12, 16, and 24 h after CLP in cirrhotic female rats fed with control diet (*n* = 28), cirrhotic female rats fed with immune enhancing diet (*n* = 28), cirrhotic male rats fed control diet (*n* = 23), and cirrhotic male rats fed with immune enhancing diet (*n* = 23). * *p* < 0.05 between male rats; ^#^*p* < 0.05 between female rats; IL-6, interleukin-6. The data are expressed as Mean ± SEM.

The changes in IL-6 following induction of sepsis among non-orchidectomized male rats and non-oophorectomized female rats are shown in Figure 2. Female rats fed immune enhancing diet had significantly lower IL-6 than the rats fed control diet at 4 h (4 ± 1 μg/mL *vs.* 42 ± 3 μg/mL). Both the male and female rats fed immune enhancing diet had significantly lower IL-6 than the rats fed control diet at 8 h (male rats: 20 ± 2 μg/mL *vs.* 30 ± 4 μg/mL; female rats: 2 ± 0 μg/mL *vs.* 8 ± 2 μg/mL). The response of IL-6 between male and female rats following immune enhancing diet feeding was similar, and they produced less pro-inflammatory cytokines after immunonutrition.

**Figure 3:**
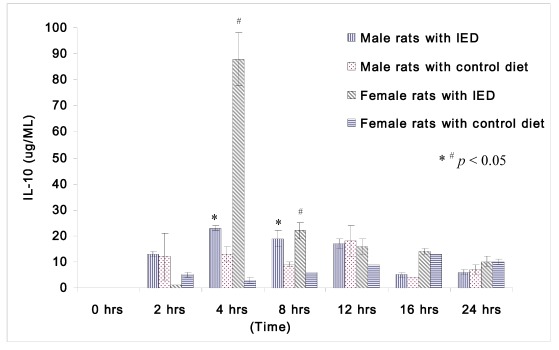
Plasma concentrations of IL-10 (μg/mL) in rats at 0, 2, 4, 8, 12, 16, and 24 h after CLP in cirrhotic female rats fed with control diet (*n* = 28), cirrhotic female rats fed with immune enhancing diet (*n* = 28), cirrhotic male rats fed with control diet (*n* = 23), and cirrhotic male rats fed with immune enhancing diet (*n* = 23). * *p* < 0.05 between male rats; ^#^ *p* < 0.05 between female rats; IL-10, interleukin-10. The data are expressed as Mean ± SEM.

The changes in IL-10 following induction of sepsis among non-orchidectomized male rats and non-oophorectomized female rats are shown in Figure 3. Both the male and female rats fed immune enhancing diet had significantly higher IL-10 than the rats fed control diet at 4 h (male rats: 23 ± 1 μg/mL *vs.* 13 ± 3 μg/mL; female rats: 88 ± 10 μg/mL *vs.* 3 ± 1 μg/mL), and at 8 h (male rats: 19 ± 3 μg/mL *vs.* 9 ± 1 μg/mL; female rats: 22 ± 3 μg/mL *vs.* 6 ± 0 μg/mL).The response of IL-10 between male and female rats following immune enhancing diet feeding was similar, and they produced higher anti-inflammatory cytokines after immunonutrition.

## 4. Discussion

The liver contains a large number of immune cells representing the innate immune system (e.g., NK cells, NKT cells, γ/δ T cells and Kupffer cells). These play a key role, not only in the host defenses against invading microorganisms and tumor formation, but also in the pathogenesis of various inflammatory liver diseases [1]. Liver fibrosis and the end-stage of liver fibrosis, cirrhosis, represent the final common pathway of virtually all kinds of chronic liver diseases [21]. Liver cirrhosis can be produced in animals with chronic infusion of CCl_4_. Chronic intoxication with CCl_4_ may result in hepatocyte damage, necrosis, inflammation and fibrosis. This spreads to link the vascular structures that feed into and drain the hepatic sinusoid, and over 8–12 weeks results in the development of cirrhosis [22].

There is not enough healthy liver tissue in a cirrhotic liver to perform the metabolic and detoxification processes that lead to impaired immunity in cirrhotic patients. Immune paralysis, defined as decreased HLA-DR expression on monocytes, indicating immune dysfunction, was found in sepsis, severe acute pancreatitis and acute liver failure. Child-Pugh class C cirrhotic patients suffer from down-regulation of HLA-DR expression. Endotoxemia contributes to this HLA-DR down-regulation [23]. The natural killer cell activity was significantly decreased in cirrhotic patients compared with normal controls and patients with chronic active hepatitis. Cirrhotic patients with Pugh’s C grade of severity of liver disease had lower natural killer cell activity. The depression of natural killer cell activity in cirrhotic patients was inversely correlated with prothrombin time ratios, and the natural killer cell activity in cirrhotic patients with hepatic encephalopathy was lower than in patients without hepatic encephalopathy. Thus, the diminished natural killer cell activity in cirrhotic patients might be related to the severity of liver damage [2].

Several specific nutrients, such as arginine, glutamine, ω-3 fatty acids and nucleotides, have been demonstrated to enhance immune function in various experimental models and clinical studies [24,25,26]. These nutritional substrates can improve the proliferation and functions of macrophages and lymphocytes thus enhancing patient cellular immunity. Arginine is essential in catabolic states in adults; it plays a fundamental role in nitrogen metabolism and polyamine synthesis [23,27,28]. Glutamine is an important energy source for enterocytes and exerts an immunomodulational effect. Glutamine-enriched enteral nutrition has been proven to reduce the incidence of sepsis in trauma patients [29,30]. Omega-3 fatty acids modulate the production of both lipid (eicosanoids) and protein (cytokines) mediators, they interact with the ω-6 fatty acids in a highly complex manner [31,32,33]. There are many studies involved trauma, surgical and critically ill patients. Surgical patients with gastrointestinal cancer were analyzed separately. The analysis demonstrated that supplementation with immunonutrition was associated with a significantly decreased incidence of infectious complications and length of hospital stay in critically ill and postoperative surgical patients with GI cancer [34].

Our data demonstrated different responses to immune enhancing diet between male and female cirrhotic rats. The mortality of cirrhotic male rats with sepsis is roughly the same as that of cirrhotic female rats, but decreased significantly after feeding with immune enhancing diet. The male rats thus need more aggressive immunomodulation to prevent higher mortality in sepsis than female rats. The mortality of cirrhotic female rats did not differ significantly between the two different diets. Immunomodulation was also important for both oophorectomized female and orchidectomized male rats. More importantly, the female oophorectomized cirrhotic rats thus required the protection of sepsis by immune enhancing diet once they lost the protection of their own sex hormones.

The female rats have the lowest levels of sex hormones during the estrous phase, and the highest levels of sex hormones in their pre-estrous phase. The estrous cycle of rats is usually 4 days/cycle [20]. As the feeding of our study takes 5 days, and the need for low levels of sex hormones before feeding, we chose female rats in a similar estrous phase at the beginning of the study and induced sepsis around their diestrous or proestrous phase when the levels of sex hormones will be high. The change of pro-inflammatory cytokines, such as IL-1β or IL-6, could explain the different responses of non-orchidectomized male and non-oophorectomized female rats following feeding of immune enhancing or non-immune enhancing diet. The above data reveal that female cirrhotic rats produce less pro-inflammatory cytokine following feeding with an immune enhancing diet, but the male rats did not. The response of IL-10 was similar in both male and female rats following feeding with an immune enhancing diet, which overcomes the higher pro-inflammatory cytokine produced by male rats than female rats. 

From our data presented here, both the non-orchidectomized cirrhotic male and non-oophorectomized female cirrhotic rats fed with immune enhancing diet displayed significantly higher IL-10 than the rats fed with non-immune enhancing diet, reflecting the important effects of the immune enhancing diet. Interleukin-10 is known as a potent anti-inflammatory cytokine, which inhibits the production of other cytokines from activated macrophages and T-helper cells [34]. Increased production of IL-10 is an important regulatory mechanism in controlling cytokine-producing cells [35]. Moreover it is rather protective, producing increased survival rates in experimentally induced sepsis [36,37,38]. Mortality reduced significantly in male rats fed with an immune enhancing diet, but the female rats displayed no significant change, with mortality remaining low regardless. This phenomenon can be explained by the fact that male rats require more aggressive immunostimulation to produce sufficient anti-inflammatory cytokines, such as IL-10, to reduce their mortality from sepsis. 

## 5. Conclusion

In conclusion, immunonutrition was more beneficial for cirrhotic male rats than female rats following sepsis. Though orchidectomy was not found to be more advantageous for the normal male rats in sepsis, immunonutrition seemed to be as important as sex hormone for female rats in sepsis. 

## Implications

Our previous publication [9] had a pilot study to show evidence about gender-different responses after immunonutrition in sepsis. Now, this study again reveals similar evidence in a cirrhotic condition, which will provide more suggestions for the clinical use of immunonutrition.
